# The Long-Term Effect of COVID-19 Disease Severity on Risk of Diabetes Incidence and the Near 1-Year Follow-Up Outcomes among Postdischarge Patients in Wuhan

**DOI:** 10.3390/jcm11113094

**Published:** 2022-05-30

**Authors:** Jun Zhang, Tingting Shu, Rui Zhu, Fengwen Yang, Boli Zhang, Xuefeng Lai

**Affiliations:** 1 Department of Intensive Care Unit, Wuhan Hospital of Traditional Chinese Medicine, Wuhan 430022, China; 15327355513@163.com (J.Z.); stt200126@163.com (T.S.); 2 Department of Traditional Chinese Medicine, Union Hospital, Tongji Medical College, Huazhong University of Science and Technology (HUST), Wuhan 430022, China; zhurui19830108@163.com; 3 State Key Laboratory of Component-Based Chinese Medicine, Tianjin University of Traditional Chinese Medicine, Tianjin 300193, China; 13682027022@163.com (F.Y.); zhangbolipr@163.com (B.Z.); 4 Department of Occupational and Environmental Health, Key Laboratory of Environment and Health, Ministry of Education & State Environmental Protection Key Laboratory of Health Effects of Environmental Pollution, School of Public Health, Tongji Medical College, Huazhong University of Science and Technology (HUST), Wuhan 430030, China

**Keywords:** COVID-19, sequelae, follow-up, long-term effects, incident diabetes

## Abstract

We assessed the nearly 1-year health consequences following discharge and related risk factors of COVID-19 infection and further explored the long-term effect of COVID-19 disease severity on the risk of diabetes incidence. This prospective study included 248 COVID-19 patients discharged from Wuhan Hospital of Traditional Chinese Medicine who were followed up between 1 March and 10 June 2021. Logistic regression models were used to evaluate risk factors. The top ten symptoms were shortness of breath (30.3%), sore or dry throat (25.7%), cough (23.2%), expectoration (23.2%), body pain (22.3%), chest tightness (20.8%), palpitations (17.8%), sleep difficulties (17.0%), fatigue (16.6%), and anxiety (15.3%). Hypertension was associated with fatigue (OR = 2.51, 95% CI: 1.08, 5.80), shortness of breath (OR = 2.34, 95% CI: 1.16, 4.69), palpitations (OR = 2.82, 95% CI: 1.26, 6.31), expectoration (OR = 2.08, 95% CI: 1.01, 4.30), and sore or dry throat (OR = 2.71, 95% CI: 1.30, 5.65). Diabetes was associated with palpitations (OR = 3.22, 95% CI: 1.18, 8.81). Critical illness was associated with an increased risk of diabetes incidence after discharge (OR = 2.90, 95% CI: 1.07, 7.88), which seemed more evident in males. Long COVID-19 symptoms were common at 1-year postdischarge; hypertension and diabetes could be projected as potential risk factors. We are among the first researchers to find that critical illness is associated with incident diabetes after discharge.

## 1. Introduction

Coronavirus disease 2019 (COVID-19), caused by severe acute respiratory syndrome coronavirus 2 (SARS-CoV-2), has been a public health emergency and a cause of huge disease burden worldwide since the outbreak in December 2019. As of 25 January 2022, the pandemic has led to more than 328 million confirmed cases and more than 5.5 million deaths globally.

As the epidemic progresses, post acute sequelae after recovery have become another major concern. It has been reported that up to 75% of COVID-19 patients have at least one persistent symptom a few months after discharge, of which the most frequently reported symptoms include chronic fatigue and shortness of breath [1]. More recently, several studies have reported one-year outcomes [2,3,4,5,6,7,8,9,10,11]; however, many of them were limited to small samples with no more than 120 patients [8,9] or included only lung function and CT results [6]. Few studies have investigated the associated risk factors for each individual symptom or included laboratory results such as inflammation markers and cytokines [2]. Therefore, the long-term health outcomes of recovery remain controversial and largely unknown. Addressing the pathophysiology of sequelae after COVID-19 is crucial for the development of treatment and prevention of poor outcomes amongCOVID-19 survivors.

On the other hand, except for the recovery outcomes, there is increasing concern about the long-term effect of COVID-19 on common chronic diseases, such as incident diabetes mellitus [12,13]. Previous studies have also indicated that severe COVID-19 may lead to worsening of hyperglycemia through increased insulin resistance and reduced β-cell secretory function [13,14,15]. However, few studies have investigated whether COVID-19 severities were associated with the long-term risk of incident diabetes.

In the current study, we aimed to evaluate the prevalence of persistent symptoms, identify the most common symptoms and related potential risk factors, and explore the associations of COVID-19 severity with incident diabetes in patients one year after discharge.

## 2. Materials and Methods

### 2.1. Study Design and Population

We recruited 263 adult patients who were laboratory-confirmed COVID-19 and discharged from designated hospitals in Wuhan between 10 January and 1 April 2020. All the enrolled patients met the discharge criteria in the Diagnosis and Treatment Protocol issued by the National Health Commission. All invited patients were tested negative by real-time reverse transcriptase-polymerase chain reaction (RT-PCR) assay from throat swab specimens. We excluded patients who declined to participate; finally, a total of 248 patients were enrolled in the study.

### 2.2. Follow-Up and Data Collection

The follow-up was conducted from 1 March to 10 June 2021. The disease severity of acute COVID-19 was defined by the World Health Organization guidelines for COVID-19 [16,17]. Critical illness was defined by the following criteria: (1) respiratory failure requiring mechanical ventilation, (2) shock, and (3) complications with other organ failures that required monitoring and treatment in an intensive care unit (ICU). Patients were interviewed via telephone by trained physicians using a uniform questionnaire, including demographics (age, sex, smoking, drinking), clinical characteristics (self-reported comorbidities, treatment information), and detailed self-reported symptoms. Participants who ever smoked or were currently smoking at least one cigarette per day for more than half a year were defined as smokers; those who ever drank or were currently drinking at least one time per week for more than half a year were considered drinkers. We combined ever/current smokers or drinkers because there were only 11 smokers and 28 drinkers. Hypertension was defined as individuals with a self-reported physician diagnosis of hypertension, blood pressure ≥140/90 mmHg, or current use of antihypertensive medication. Prevalent diabetes was defined as a self-reported physician diagnosis of diabetes, fasting glucose level ≥7.0 mmol/L, or taking oral hypoglycemic medication or insulin. We combined cardiovascular disease (*n* = 12), cerebrovascular disease (*n* = 4), malignant tumor (*n* = 7), chronic pulmonary (*n* = 8), liver (*n* = 9), and kidney (*n* = 4) diseases together as a new variable of the other chronic diseases to be adjusted in the models because these small cases of comorbidities could not be separately and validly adjusted. All reported symptoms were carefully confirmed to exclude any potential symptoms that existed before the patient was infected with COVID-19. All patients were invited to receive a physical examination, laboratory tests, and chest high-resolution CT (HRCT). All data were double-entered and checked by another researcher.

### 2.3. Definition of Incident Diabetes

To control any potential reverse causal association, we excluded diabetes that occurred before infection with SARS-Cov-2 or during the initial hospitalization for treatment of COVID-19. That is, we defined the incidence of diabetes as newly diagnosed hyperglycemia after discharge (fasting blood glucose > 7.00 mmol/L) during this follow-up. All patients underwent at least two fasting blood glucose tests on different days, according to international guidelines.

### 2.4. Statistical Analysis

Continuous variables and categorical variables are presented as medians (interquartile ranges, IQR) and counts (%), respectively. To understand the importance of critical illness on long-term health outcomes, participants were categorized into two groups according to whether they were critically ill or not during their initial hospital stay. The comparison of baseline characteristics and laboratory results during follow-up across two groups of participants was performed by *t*-test or Mann–Whitney U test, and χ^2^ test, Fisher’s exact test, as appropriate. Multivariable adjusted logistic regression models were applied to estimate the odds ratios (ORs) and 95% CIs for the associations of potential risk factors with the top ten symptoms. Confounders included in the above models were age, sex, body mass index (BMI), smoking, drinking, hypertension, diabetes, and other chronic comorbidities, including cardiovascular diseases, cerebrovascular diseases, malignancy, chronic pulmonary disease, chronic liver disease, chronic kidney disease, and interleukin-8 (IL-8). We also used logistic regression to explore associations of disease severity with risk of diabetes incidence after excluding pre-existing diabetes and adjusted the same covariates. Stratification analysis across sex was conducted, and potential interactions were tested by adding a cross product term. All statistical analyses used SAS software (version 9.4; SAS Institute Inc., Cary, NC, USA) and R software (version 4.0.3; the R foundation, The University of Auckland, Newzealand, free access to URLS: https://mirror.lzu.edu.cn/CRAN/, accessed on 12 June 2020). All *p* values for the tests were two-sided, and *p* values < 0.05 were considered statistically significant.

## 3. Results

### 3.1. Baseline Characteristics

The demographic and clinical characteristics of the participants are shown in Table 1. Of the 248 available for the near one-year follow-up, there were 112 (45.2%) men and 136 (54.8%) women. The median age of the enrolled participants was 61.0 (54.0–68.0) years, and critically ill patients were older than the counterpart group. The median body mass index (BMI) was 25.1 (23.2, 27.3). Eleven patients (4.4%) and twenty-eight patients (11.3%) were or had ever been smokers or drinkers, respectively. The proportions of smokers and drinkers (past or present) were similar across the two groups. The most common comorbidity was hypertension (63 patients, 25.4%), followed by diabetes (25 patients, 10.1%), cardiovascular disease (12 patients, 4.8%), liver disease (9 patients, 3.6%), and chronic lung disease (8 patients, 3.2%). There was no difference in comorbidity prevalence between critically ill patients and noncritically ill patients, except for a higher prevalence of chronic lung disease in noncritically ill patients.

### 3.2. One Year Outcomes after Hospital Discharge

As shown in Table 2, the top ten symptoms were shortness of breath (30.3%), sore or dry throat (25.7%), cough (23.2%), expectoration (23.2%), body pain (22.3%), chest tightness (20.8%), palpitations (17.8%), sleep difficulties (17.0%), fatigue (16.6%), and anxiety (15.3%). Furthermore, the most common abnormal CT manifestations were ground-glass opacity (GGO; 65 patients, 33.9%), followed by consolidation (30 patients, 15.6%). The proportion of other abnormal CT manifestations was lower than 5% and included interlobular septal thickening, Subpleural line, crazy-paving pattern, and reticular pattern. 

In addition, Table 3 shows the laboratory results. All participants showed healthy blood cell counts, including white blood cells, lymphocytes, neutrophils, red blood cells, and platelets. Similarly, the median levels of most biochemistry indices were within normal ranges, such as alanine aminotransferase (ALT), aspartate aminotransferase (AST), creatinine, triglyceride, total cholesterol, and fasting blood glucose. However, we found that IL-8 was still very high (44.4 pg/mL) though other cytokines were within the normal ranges whether patients were critically ill or not. Critically ill patients still have relatively higher interleukin-6 (IL-6) and creatinine levels (all *p* < 0.05).

### 3.3. Risk Factors of Post Sequelae One Year after Discharge

Table 4 shows potential risk factors of the top ten post sequelae one year after discharge. After multivariable adjustment, hypertension (OR = 2.51, 95% CI: 1.08, 5.80) and IL-8 (OR = 2.40, 95% CI: 1.12, 5.17) were associated with a higher risk of fatigue. Female sex (OR = 1.94, 95% CI: 1.01, 3.77) and hypertension (OR = 2.34, 95% CI: 1.16, 4.69) were associated with a higher risk of shortness of breath. Hypertension was also associated with a higher risk of palpitations (OR = 2.82, 95% CI: 1.26, 6.61), expectoration (OR = 2.08, 95% CI: 1.01, 4.30) and sore or dry throat (OR=2.71, 95% CI: 1.30, 5.65). Diabetes was related to palpitation (OR = 3.22, 95% CI: 1.18, 8.81). Age (OR = 1.04, 95% CI: 1.01, 1.08) and female sex (OR = 2.52, 95% CI: 1.04, 6.11) were associated with sleep difficulties, and female sex was also related to sore or dry throat (OR = 2.46, 95% CI: 1.21, 4.98).

### 3.4. Associations between COVID-19 Severity and Risk of Diabetes Incidence

As shown in Table 5, a total of 19 occurrences of diabetes (8.6%) were observed one year after discharge. Multivariable logistic regression showed that critical illness was associated with a higher risk of diabetes incidence one year after discharge (OR = 2.90, 95% CI: 1.07, 7.88). Furthermore, stratification analysis indicated that this association was more evident in males (OR = 5.70, 95% CI: 1.46, 22.15) though no significant interaction was observed.

## 4. Discussion

This longitudinal study systematically assessed the long-term health outcomes of COVID-19 patients nearly one year after discharge from Wuhan Chinese Medicine Hospital. We found that 51 patients (20.6%) reported at least one persistent or emerging symptom, and the top ten symptoms were lassitude, fatigue, sweating, palpitation, chest tightness, shortness of breath, cough, expectation, anxiety, and sleep difficulties. Interestingly, hypertension and diabetes were associated with higher risks for the above postinfection symptoms. Severity was associated with the fibrotic lesion, and IL-8 was also related to fatigue. More importantly, to our knowledge, this study was among the first to evaluate the long-term effects of disease severity on the risk of developing diabetes. We found that being critically ill during the initial hospitalization was associated with a higher risk of diabetes incidence nearly one year after discharge, and this association seemed to be more evident in males.

Our study revealed the top ten postinfection symptoms, which covered the most common post sequelae reported for 1-year healthy outcomes in previous studies. An earlier study of 1276 discharged COVID-19 patients reported that fatigue or muscle weakness, sleep difficulties, palpitations, and joint pain were the most common symptoms at 1-year follow-up [2]. Another study of 1095 patients also reported that fatigue, sweating, chest tightness, anxiety, and myalgia were the most common symptoms [7]. More recently, a multicenter study of 1233 COVID-19 survivors found the top ten post sequelae included fatigue, sweating, chest tightness, anxiety, myalgia, cough, palpitation, shortness of breath, expectoration, and dizziness [10]. More importantly, we found that hypertension and diabetes are crucial risk factors for systemic sequelae such as lassitude and fatigue, cardiovascular sequelae such as palpitation and chest tightness, and respiratory sequelae including shortness of breath, cough, and expectoration. This finding may indicate that strict management of blood pressure and fasting glucose could greatly contribute to relieving symptoms and promoting early recovery. In fact, hypertension and diabetes have been demonstrated to be associated with higher severity incidence and hospitalization mortality [18,19]. Though the mechanism is not clear, it is plausible that elderly patients with long-term hypertension and diabetes may develop cardiovascular disease and pulmonary hypertension and then induce cardiovascular and respiratory symptoms [20,21,22]. Patients with diabetes tend to suffer chronic inflammation [23], and, consequently, COVID-19 patients with hypertension and diabetes may experience a prolonged duration of the above symptoms. In addition, our laboratory results showed that IL-8 was still very high though other cytokines such as IL-1β, IL-6, and IL-10 were all within normal range. Multivariable logistic regression further indicated that high IL-8 is associated with a higher prevalence of fatigue and lassitude. This finding may provide a clue for relieving fatigue sequelae and should be further confirmed in a future follow-up study.

On the other hand, our study focused on the long-term effects of COVID-19 severity on the risk of diabetes incidence. To our knowledge, we are among the first to find that critical illness may increase the long-term risk of diabetes morbidity, and this association was more evident in males. An earlier study reported a bidirectional relationship between COVID-19 and diabetes [12]. More recently, a meta-analysis of eight studies with more than 3700 patients showed a pooled proportion of 14.4% for newly diagnosed diabetes in hospitalized COVID-19 patients during the acute phase [13], which partly supported our findings. Furthermore, many studies have reported that severe complications, such as diabetic ketoacidosis and hyperosmolarity, are common in COVID-19 patients with diabetes [24,25], especially in new-onset diabetes [26]. Results from two ecological analyses based on the DPV (Diabetes-Patienten-Verlaufsdokumentation) database observed no short-term but a long-term increase in the incidence of type 1 diabetes in children and adolescents in Germany during the COVID-19 pandemic, with a delay in the peak incidence of type 1 diabetes by 3 months after the peak COVID-19 incidence [27,28]. These two studies provided evidence of the indirect effects of the pandemic on type 1 diabetes incidence in youngsters. More importantly, a retrospective study in the US also showed that persons aged <18 years with COVID-19 are more likely to receive a new diabetes diagnosis >30 days after infection than those without COVID-19 and those with prepandemic acute respiratory infections [29]. Another large retrospective study in Germany further showed that COVID-19 confers an increased risk for type 2 diabetes compared with matched controls [30]. Collectively, these retrospective studies, but with control groups, supported our results.

We observed a sex difference in the relationship between critical illness and subsequent risk of diabetes incidence among postdischarge COVID-19 patients. Current evidence suggests that men have a higher risk of COVID-19 infection, hospitalization, disease severity, ICU admission, and death than women [31], which may partly support our result about sex differences. Though the mechanism was unclear, genetic/epigenetic factors, sex differences in the immune response [32,33], androgens-mediated ACE2 expression [34,35], and gut microbiome-mediated immune modulation [36] may be reasons for sex-based differences. Another alternative explanation is that the observed difference may be attributed to limited incident cases and insufficient statistical power for female patients. Our results suggested that sex differences could be considered in the future surveillance of the long-term effects of COVID-19. Future large-scale prospective studies are warranted to confirm our findings.

The pathophysiological mechanisms are still not clear; however, the current evidence indicates that the direct effect of the virus on pancreatic β-cells through the angiotensin-converting enzyme-2 (ACE2) receptor [14,37,38] and prolonged inflammation with immune dysregulation [39,40] may be responsible for COVID-19 related diabetes [15]. The current literature indicates that COVID-19 may have not only temporal effects on raising glucose but also have long-term diabetogenic effects of increasing the future risk of full-blown diabetes occurrence [13,15,41,42]. Our long-term results also further provided evidence that newly diagnosed diabetes in COVID-19 patients may not be entirely attributed to the stress response or glucocorticoids treatment. Large prospective studies with longer follow-ups are warranted to confirm our findings. A global registry of patients with COVID-19-related diabetes (covidiab.e-dendrite.com, accessed on 12 June 2020) has been established to address the issues associated with the bidirectional relationship between COVID-19 and newly diagnosed diabetes [12]. Our study reported the top ten postinfection symptoms and found hypertension and diabetes are vital risk factors for common sequelae, which may add new clues for the promotion of early recovery through better control of blood pressure and glucose. We are among the first to report a potential long-term effect of COVID-19 severity on the increased risk of diabetes incidence. Our findings have important public health implications for COVID-19 survivors. However, our study has several limitations. First, self-reported symptoms may overestimate or underestimate the prevalence of the post sequelae, although we performed the interview with uniformly trained physicians and carefully confirmed each symptom with each patient. There was also no standardization for some prognosticating parameters, such as the combined variable of the other chronic diseases. Second, the absence of a non-COVID-19 control group precluded the comparison of postinfection symptom prevalence with non-COVID-19 individuals. In addition, the present analysis did not support any independent or dependent risk factors for post sequelae or incident diabetes because of a lack of a control group, so it can be projected as a risk factor only. However, the latest two large-scale retrospective studies with well-controlled groups in the US [29] and Germany [30] reinforced the reliability of our results and thus enhanced the significance. Third, the generalizability of the results may be limited by the single-center design and location of a single city in China and the relatively small sample size. Diverse groups from various palaces in the county would have added value to the results. Fourth, we did not distinguish between type 1 and type 2 diabetes, though we speculated that most diabetes that occurred postinfection in our study was likely to be type 2 diabetes given the age of the participants (>60 years old). Fifth, the assessed parameters were not available at discharge and at additional time points during the follow-up, which affected our ability to analyze the hyperglycemia changes over time, thus obtaining a better understanding of the incidence of diabetes and the underlying effect. Finally, our results on the long-term effects of COVID-19 severity on the increased risk of diabetes should be confirmed in a future large prospective study with a control group of non-COVID-19 patients and a longer follow-up.

## 5. Conclusions

Our study indicated that the most common postinfection symptoms were lassitude, fatigue, sweating, palpitation, chest tightness, short breath, cough, expectoration, anxiety, and sleep difficulties. We also found that hypertension and diabetes may be projected as potential risk factors for the postinfection symptoms. We are among the first to find that critical illness during initial hospitalization is associated with a higher risk of incidence of diabetes nearly one year after discharge. Large prospective studies are warranted to validate our results.

## Figures and Tables

**Table 1:** Baseline characteristics according to disease severity.

	Total (*n* = 248)	Critically Ill Patients		*p* Value
		No (*n* = 171)	Yes (*n* = 77)	
Age	61.0 (54.0, 68.0)	60.0 (52.0, 67.0)	64.5 (57.0, 70.0)	0.06
Sex				0.27
Male	112 (45.2%)	73 (42.7%)	39 (50.7%)	
Female	136 (54.8%)	98 (57.3%)	38 (49.3%)	
BMI, kg/m²	25.1 (23.2, 27.3)	25.4 (23.3, 27.3)	25.2 (228, 27.1)	
Smoking	11/248 (4.4%)	7/171 (4.1%)	4/77 (5.2%)	0.74
Drinking	28/248 (11.3%)	21/171 (12.3%)	7/77 (9.1%)	0.46
**Comorbidities**				
Hypertension	63/248 (25.4%)	40/171 (23.4%)	23/77 (29.9%)	0.28
Diabetes	25/248 (10.1%)	16/171 (9.4%)	9/77 (11.7%)	0.57
Cardiovascular disease	12/248 (4.8%)	8/171 (4.7%)	4/77 (5.2%)	0.86
Cerebrovascular disease	4/248 (1.6%)	2/171 (1.2%)	2/77 (2.6%)	0.59
Malignant tumor	7/248 (2.8%)	4/171 (2.3%)	3/77 (3.9%)	0.68
Chronic lung disease	8/248 (3.2%)	2/171 (1.2%)	6/77 (7.8%)	0.01
Liver disease	9/248 (3.6%)	8/171 (4.7%)	1/77 (1.3%)	0.28
Chronic kidney disease	4/248 (1.6%)	3/171 (1.8%)	1/77 (1.3%)	0.79

Notes: Data are *n* (%), *n*/N (%), or median (IQR).

**Table 2:** Symptoms and chest CT results of follow-up according to severity scale.

	Total (*n* = 248)	Critically Ill Patients		*p* Value
		No (*n* = 171)	Yes (*n* = 77)	
**Symptoms**				
Fever	10/241 (4.2%)	5/167 (3.0%)	5/74 (6.8%)	0.18
Headache	26/241 (10.8%)	17/167 (10.2%)	9/74 (12.2%)	0.65
Body pain	53/241 (22.0%)	34/167 (20.4%)	19/74 (25.7%)	0.38
Shortness of breath	73/241 (30.3%)	53/167 (31.7%)	20/74 (27.0%)	0.46
Chest tightness	50/241 (20.8%)	33/167 (19.8%)	17/74 (23.0%)	0.57
Palpitations	43/241 (17.8%)	29/167 (17.4%)	14/74 (18.9%)	0.77
Fatigue	40/241 (16.6%)	29/167 (17.4%)	11/74 (14.9%)	0.63
Lassitude	27/241 (11.2%)	17/167 (10.2%)	10/74 (13.5%)	0.45
Anxiety	36/236 (15.3%)	25/164 (15.2%)	11/72 (15.3%)	0.99
Sleep difficulties	40/236 (17.0%)	32/164 (19.5%)	8/72 (11.1%)	0.11
Cold limbs	18/241 (7.5%)	12/167 (7.2%)	6/74 (8.1%)	0.80
Smell disorder	11/241 (4.6%)	10/167 (6.0%)	1/74 (1.4%)	0.18
Taste disorder	16/241 (6.6%)	10/167 (6.0%)	6/74 (8.1%)	0.58
Cough	56/241 (23.2%)	41/167 (24.6%)	15/74 (20.3%)	0.47
Expectoration	56/241 (23.2%)	39/167 (23.4%)	17/74 (23.0%)	0.95
Nausea or vomiting	7/241 (2.9%)	5/167 (3.0%)	2/74 (2.7%)	1.00
Decreased appetite	12/241 (5.0%)	10/ 167 (6.0%)	2/74 (2.7%)	0.35
Sore or dry throat	62/241 (25.7%)	45/ 167 (27.0%)	17/74 (23.0%)	0.52
Nasal obstruction	6/236 (2.5%)	5/164 (3.1%)	1/72 (1.4%)	0.67
**Chest CT**				
Ground glass opacity	65/192 (33.9%)	46/125 (36.8%)	19/67 (28.4%)	0.24
Consolidation	30/192 (15.6%)	21/125 (16.8%)	9/67 (13.4%)	0.54
Interlobular septal thickening	5/192 (2.6%)	2/125 (1.6%)	3/67 (4.5%)	0.34
Subpleural line	7/192 (3.7%)	5/125 (4.0%)	2/67 (3.0%)	1.00
Crazy-paving pattern	3/192 (1.6%)	1/125 (0.8%)	2/67 (3.0%)	0.28
Reticular pattern	5/192 (2.6%)	2/125 (1.6%)	3/67 (4.5%)	0.35

Notes: Data are *n*/N (%) or median (IQR). All models are adjusted for age, sex, and comorbidities, including hypertension, diabetes, and other chronic diseases (cardiovascular disease, cerebrovascular disease, chronic liver disease, chronic kidney disease, cancer, and gallbladder disease).

**Table 3:** Laboratory results at follow-up according to severity scale.

Covariates (Normal Range)	Total (*n* = 248)	Critically Ill Patients		*p* Value
		No (*n* = 171)	Yes (*n* = 77)	
**Complete blood count**				
WBC, ×10^9^/L (4–10)	5.4 (4.4, 6.3)	5.4 (4.4, 6.2)	5.1 (4.4, 6.4)	0.97
Neutrophils, ×10^9^/L (1.8–6.3)	3.0 (2.5, 3.8)	3.0 (2.5, 3.7)	3.0 (2.4, 3.9)	0.62
Lymphocytes, ×10^9^/L (1.1–3.2)	1.6 (1.4, 2.0)	1.7 (1.4, 2.0)	1.5 (1.3, 1.9)	0.05
Monocytes, ×10^9^/L (0.1–0.6)	0.4 (0.3, 0.4)	0.4 (0.3, 0.4)	0.4 (0.3, 0.5)	0.97
RBC, ×10^12^/L (3.5–6.0)	4.6 (4.3, 4.9)	4.6 (4.3, 4.9)	4.6 (4.3, 4.8)	0.49
Hemoglobin, g/L (115–150)	140.0 (133.0, 149.0)	140.0 (133.0, 148.0)	140.0 (134.0, 149.0)	0.74
Platelets, ×10^9^/L (125–350)	210.5 (179.5, 250.0)	209.0 (180.0, 250.0)	213.0 (179.0, 247.0)	0.98
NLR	1.9 (1.5, 2.3)	1.8 (1.4, 2.3)	1.9 (1.5, 2.4)	0.06
**Lymphocyte subsets**				
CD3+, (58–84%)	65.0 (57.8, 71.5)	65.0 (57.7, 71.5)	65.1 (58.2, 71.2)	0.67
CD4+, (25–51%)	37.3 (32.1, 42.9)	38.0 (31.8, 43.7)	37.1 (32.3, 41.4)	0.37
CD8+, (14–39%)	24.8 (19.8, 30.8)	24.2 (20.0, 30.6)	25.3 (18.9, 32.3)	0.40
NK cell, (3–30%)	22.9 (16.1, 30.4)	22.9 (15.8, 30.3)	23.3 (16.9, 31.1)	0.57
B lymphocyte, (4–18%)	9.6 (7.0, 12.8)	9.9 (7.2, 13.0)	9.4 (6.7, 12.1)	0.19
CD4+/CD8+ ratio (0.41–2.72)	1.5 (1.1, 2.0)	1.5 (1.1, 2.0)	1.5 (1.1, 2.0)	0.40
**Cytokines**				
IL-1β, pg/mL (0.1–5)	5.0 (5.0, 5.0)	5.0 (5.0, 5.0)	5.0 (5.0, 5.0)	0.51
IL-6, pg/mL (0.1–2.9)	3.1 (2.2, 4.2)	2.9 (2.1, 4.1)	3.5 (2.6, 4.9)	**0.01**
IL-8, pg/mL (0.1–10)	44.4 (19.2, 115.5)	41.9 (17.4, 113.0)	44.5 (28.1, 119.0)	0.19
IL-10, pg/mL (0.1–5)	5.0 (5.0, 5.0)	5.0 (5.0, 5.0)	5.0 (5.0, 5.0)	0.41
**Biochemical indices**				
Triglycerides, mmol/L (0.5–1.7)	1.4 (1.0, 2.1)	1.4 (1.0, 2.1)	1.4 (1.1, 2.1)	0.97
Total cholesterol, mmol/L (3.0–5.7)	5.1 (4.4, 5.7)	5.0 (4.4, 5.7)	5.1 (4.5, 5.8)	0.76
High density lipoprotein, mmol/L (0.9–1.8)	1.5 (1.2, 1.7)	1.5 (1.3, 1.7)	1.4 (1.2, 1.7)	0.24
Low density lipoprotein, mmol/L (0–3.12)	2.9 (2.4, 3.5)	2.9 (2.4, 3.4)	2.9 (2.4, 3.5)	0.77
ALT, U/L (5–35)	20.0 (15.0, 28.5)	21.0 (16.0, 30.0)	19.0 (14.0, 26.0)	0.15
AST, U/L (8–40)	23.0 (20.0, 28.0)	24.0 (20.0, 28.0)	23.0 (20.0, 27.0)	0.67
Creatinine, μmol/L (44–106)	66.0 (56.0, 79.0)	64.0 (55.0, 76.0)	70.0 (60.0, 83.0)	**0.01**
BUN, mmol/L (2.9–8.2)	5.5 (4.6, 6.6)	5.5 (4.5, 6.5)	5.5 (4.9, 6.7)	0.14
Total bilirubin, μmol/L (5.1–19)	12.0 (9.5, 14.9)	11.8 (9.5, 14.5)	12.4 (9.4, 16.8)	0.18
Creatine kinase, U/L (26–140)	100.0 (75.5, 131.5)	101.0 (74.0, 129.0)	98.0 (77.0, 134.0)	0.68
Troponin I, ng/L (<26.2)	<2.00	<2.00	<2.00	0.99
LDH, U/L (109–245)	163.0 (146.0, 176.0)	161.0 (145.0, 176.0)	166.0 (149.0, 176.0)	0.82

Notes: Data are *n*/N (%), or median (IQR), ALT: alanine aminotransferase; AST: aspartate aminotransferase; BUN: blood urea nitrogen; IL: interleukin; LDH: lactate dehydrogenase; RBC: red blood cell; WBC: white blood cell.

**Table 4:** Risk factors associated with the top ten symptoms one year after discharge.

Variables	OR (95% CI) and *p* Values for Each Post Symptom									
	Fatigue		Body Pain		Shortness of Breath		Chest Tightness		Palpitations	
age	1.00 (0.97, 1.03)	0.85	1.01 (0.99, 1.04)	0.38	0.99 (0.97, 1.01)	0.40	1.01 (0.97, 1.03)	0.89	1.00 (0.96, 1.03)	0.98
Sex, female	1.19 (0.55, 2.54)	0.66	1.36 (0.68, 2.74)	0.39	1.94 (1.01, 3.77)	0.04	1.75 (0.83, 3.68)	0.14	1.83 (0.83, 4.06)	0.14
smoking	0.68 (0.07, 6.33)	0.73	1.00 (0.19, 5.34)	1.00	3.56 (0.92, 13.83)	0.07	0.46 (0.05, 4.06)	0.49	NA	NA
drinking	0.14 (0.02, 1.20)	0.07	0.74 (0.22, 2.48)	0.63	0.75 (0.26, 2.19)	0.60	1.10 (0.32, 3.79)	0.89	0.46 (0.09, 2.34)	0.35
Critically ill	0.71 (0.32, 1.56)	0.39	1.36 (0.70, 2.64)	0.36	0.81 (0.42, 1.53)	0.51	1.27 (0.63, 2.54)	0.50	1.05 (0.49, 2.28)	0.90
Hypertension	2.51 (1.08, 5.80)	0.03	0.97 (0.45, 2.11)	0.94	2.34 (1.16, 4.69)	0.02	1.28 (0.58, 2.81)	0.54	2.82 (1.26, 6.31)	0.01
Diabetes	0.86 (0.27, 2.68)	0.79	0.89 (0.30, 2.67)	0.84	0.56 (0.20, 1.61)	0.28	2.56 (0.97, 6.77)	0.06	3.22 (1.18, 8.81)	0.02
Other chronic diseases	0.78 (0.29, 2.12)	0.62	0.96 (0.42, 2.23)	0.93	0.96 (0.45, 2.04)	0.91	0.83 (0.34, 1.99)	0.46	1.31 (0.55, 3.12)	0.54
IL-8	2.40 (1.12, 5.17)	0.02	1.09 (0.53, 2.26)	0.82	0.79 (0.39, 1.60)	0.51	0.74 (0.33, 1.64)	0.46	0.35 (0.12, 1.00)	0.06
**Variables**	**OR (95% CI) and *p* Values for Each Post Symptom**									
	**Cough**		**Expectoration**		**Sore or Dry Throat**		**Sleep Difficulties**		**Anxiety**	
Age	0.99 (0.97, 1.02)	0.52	1.02 (0.99, 1.05)	0.12	0.99 (0.96, 1.02)	0.43	1.04 (1.01, 1.08)	0.02	0.99 (0.96, 1.03)	0.66
Sex, female	1.20 (0.61, 2.38)	0.60	0.56 (0.28, 1.12)	0.10	2.46 (1.21, 4.98)	0.01	2.52 (1.04, 6.11)	0.04	1.53 (0.37, 3.50)	0.32
Smoking	2.19 (0.53, 9.06)	0.28	2.53 (0.65, 9.84)	0.18	0.32 (0.04, 2.84)	0.31	0.96 (0.10, 9.22)	0.97	1.55 (0.27, 9.10)	0.63
Drinking	0.66 (0.21, 2.06)	0.47	0.75 (0.26, 2.13)	0.58	0.69 (0.20, 2.40)	0.56	1.81 (0.52, 6.33)	0.36	0.43 (0.09, 2.12)	0.30
Critically ill	0.78 (0.39, 1.55)	0.48	0.82 (0.41, 1.64)	0.57	0.78 (0.39, 1.53)	0.46	0.54 (0.23, 1.29)	0.16	0.93 (0.41, 2.08)	0.85
Hypertension	1.70 (0.81, 3.56)	0.16	2.08 (1.01, 4.30)	0.04	2.71 (1.30, 5.65)	0.01	0.81 (0.32, 2.04)	0.65	2.25 (0.96, 5.28)	0.06
Diabetes	1.25 (0.45, 3.43)	0.67	1.93 (0.71, 5.23)	0.20	0.85 (0.31, 2.36)	0.76	0.35 (0.07, 1.72)	0.20	1.78 (0.63, 5.02)	0.28
Other chronic diseases	0.63 (0.26, 1.49)	0.29	0.88 (0.38, 2.05)	0.77	1.28 (0.59, 2.80)	0.54	1.05 (0.38, 2.91)	0.92	1.37 (0.55, 3.42)	0.50
IL-8	0.74 (0.35, 1.57)	0.43	0.60 (0.27, 1.33)	0.21	0.88 (0.42, 1.83)	0.73	2.19 (0.98, 4.93)	0.06	1.18 (0.49, 2.80)	0.72

Notes: multivariable logistic regressions were used to estimate associated risk factors for post sequelae. NA: not applicable.

**Table 5:** Associations of COVID-19 severity with risk of diabetes incidence after discharge.

	Critical Ill Patients		*p* Value	*p* for Interaction
	No	Yes		
Total patients				
Incidents/patients (%)	9/153 (5.9%)	10/68 (14.7%)		
OR (95% CI)	Ref.	2.90 (1.07, 7.88)	0.04	
Sex				0.14
Male				
Incidents/patients (%)	4/65 (6.2%)	8/37 (21.6%)		
OR (95% CI)	Ref.	5.70 (1.46, 22.15)	0.01	
Female				
Incidents/patients (%)	5/88 (5.7%)	2/31 (6.5%)		
OR (95% CI)	Ref.	0.84 (0.12, 6.00)	0.86	

Notes: Models adjusted for age, sex, smoking, drinking, hypertension, and other chronic comorbidities, including cardiovascular disease, cerebrovascular disease, chronic liver disease, chronic kidney disease, cancer, chronic pulmonary disease, and interleukin-8. The subgroup analysis adjusted the same variables except for the stratification variable.

## Data Availability

The datasets generated and analyzed during the current study are available from the corresponding author on reasonable request.
